# Synthetic redesign of plant lipid metabolism

**DOI:** 10.1111/tpj.13172

**Published:** 2016-06-20

**Authors:** Richard P. Haslam, Olga Sayanova, Hae Jin Kim, Edgar B. Cahoon, Johnathan A. Napier

**Affiliations:** ^1^ Biological Chemistry and Crop ProtectionRothamsted Research, West CommonHarpendenAL5 2JQUK; ^2^ Centre for Plant Science Innovation and Department of BiochemistryUniversity of Nebraska‐LincolnLincolnNE68588USA

**Keywords:** fatty acid metabolism, metabolic engineering, plant biotechnology, predictive manipulation, oil crops

## Abstract

Plant seed lipid metabolism is an area of intensive research, including many examples of transgenic events in which oil composition has been modified. In the selected examples described in this review, progress towards the predictive manipulation of metabolism and the reconstitution of desired traits in a non‐native host is considered. The advantages of a particular oilseed crop, *Camelina sativa*, as a flexible and utilitarian chassis for advanced metabolic engineering and applied synthetic biology are considered, as are the issues that still represent gaps in our ability to predictably alter plant lipid biosynthesis. Opportunities to deliver useful bio‐based products via transgenic plants are described, some of which represent the most complex genetic engineering in plants to date. Future prospects are considered, with a focus on the desire to transition to more (computationally) directed manipulations of metabolism.

## Significance Statement

In this review we advance the oilseed plant *Camelina sativa* as a useful platform for metabolic engineering of lipids, and propose that a new paradigm of predictive manipulation will exceed progress made thus far with iterative approaches.

## Introduction

Plant seeds are a reservoir of energy‐dense molecules, including oils enriched in triacylglycerols (TAGs) that serve as the primary storage form of fatty acids. These TAGs and their component fatty acids not only provide a major dietary calorie source and media for food preparation, but also serve as a renewable source of hydrocarbons for biofuels and industrial applications. Although our knowledge of plant lipid metabolism and its regulation is not complete, the basic pathways of fatty acid biosynthesis, modification and assembly into triacylglycerols are well characterized. With this information and the wealth of genetic diversity for synthesis of novel fatty acids and storage oils, seeds offer a ‘palette’ and platform for the design and tailoring of biochemical pathways to generate diverse nutritional and industrial oils not currently found in oilseed crops. As tools for synthetic biology and information from large‐scale systems biology studies emerge, it is possible to rapidly assemble novel pathways in oilseed crops and achieve predictable outcomes.

For the purpose of this review, synthetic biology will be defined as the design and engineering of biologically based parts and novel activities to facilitate the redesign of existing, natural biological systems. The application of synthetic biology for metabolic engineering of plants has lagged behind synthetic biology‐based engineering of microbial systems due in part to the challenges and long time‐frames associated with obtaining transgenic plants and the imprecision and lack of predictability associated with random insertion of transgenes into the host plant genome. However, seed lipid metabolism represents a good system for building and developing synthetic biology tools for metabolic engineering because lipid biosynthetic pathways are generally well characterized, analytical protocols exist for rapid measurement of fatty acid and lipid products and lipidomic methodologies are available to assess global changes in lipid and biosynthetic intermediates for iterative improvement of engineering strategies. Concomitantly an oilseed chassis has emerged that is capable of hosting the new functions necessary to manufacture novel molecules. The oilseed plant *Camelina sativa* is both a model system and a crop platform for lipid metabolic engineering because of its close genetic relation to Arabidopsis (both being members of the family Brassicaceae), its simple, non‐labour‐intensive transformation system and its relatively short life cycle.

The fatty acid and lipid biosynthetic pathways of plants form a biochemical chassis upon which strategies for the production of novel molecules can be synthetically designed. Although questions about the regulation and biochemical architecture of enzymes in these pathways remain, the basic framework of fatty acid and lipid biosynthesis in plants is well characterized. New enzymes can be introduced from other species or rationally designed to alter carbon flux through these pathways, producing compounds of high value that are absent or typically found in low levels in oilseed crops. The aim of this review is to discuss the emergence of *C. sativa* as a platform for the production of bioproducts, outline the metabolic context and then, through a series of case studies, examine how lipid synthesis in seed oils can be redesigned to enable the accumulation of novel fatty acids that have beneficial functional groups or properties.

## An Oilseed Chassis For Lipid Synthetic Design

Camelina (*C. sativa*), an oilseed crop of the Brassicaceae, has been identified as a promising new crop for oil production due to its adaptability to temperate growing regions, low input requirements, a short crop cycle and disease resistance (Zanetti *et al*., 2013). In terms of performance, *C. sativa* has an acceptable yield (levels over 3000 kg ha^−1^ have been achieved in favourable environments) and an oil content of about 40% (Vollmann and Eynck, 2015). However, beyond its agronomic competitiveness, *C. sativa* has been recruited by researchers as a platform for the production of tailored oils (Collins‐Silva *et al*., 2011). *Camelina sativa* oil is high in the polyunsaturated fatty acids α‐linolenic acid (18:3) and linoleic acid (18:2) (substrates for nutritionally important long‐chain omega‐3 fatty acids) and low in erucic acid (22:1), a fatty acid that has been linked to heart disease (Iskandarov *et al*., 2014). This desirable fatty acid composition forms the basis for the development of nutritionally enhanced oils. The utility of *C. sativa* as a chassis for synthetic pathway design can be ascribed to: (i) the ease with which it can be transformed via *Agrobacterium* floral vacuum infiltration (Lu and Kang, 2008), and (ii) the genetic similarity between *C. sativa* and the well‐studied model plant Arabidopsis. *Camelina sativa* shares many (>90%; Nguyen *et al*., 2013; Kagale *et al*., 2014) of the genes involved in lipid metabolism with the genetic model plant Arabidopsis. It has very low outcrossing levels ranging between 0.01% and 0.28% (Walsh *et al*., 2012) and fully developed tools for breeding and molecular trait improvement due to a high sequence identity with Arabidopsis. The sequencing of the *C. sativa* genome (Kagale *et al*., 2014) and studies of the seed transcriptome (Nguyen *et al*., 2013) have made the rational design of lipid pathways in *C. sativa* more reliable and predictable. Moreover, the expanded lipid gene family in *C. sativa* (217% compared with Arabidopsis; Kagale *et al*., 2014), provides greater diversity in enzyme expression and substrate specificity; all of which permit the effective expression and integration of novel heterologous pathways. Many of the shortcomings associated with model species, discussed later, can be overcome with *C. sativa*, as it has the ability to be both an experimental model system and recognized oilseed crop. Together these features have enabled the rapid evaluation of synthetic design prototypes – multiple gene expression and trait stacking – leading to the step‐wise, iterative improvement of metabolic strategies for lipid pathway design in *C. sativa*. In this article we will provide several examples of such approaches.

## Metabolic Context: Seed Oil Synthesis

**Figure 1:**
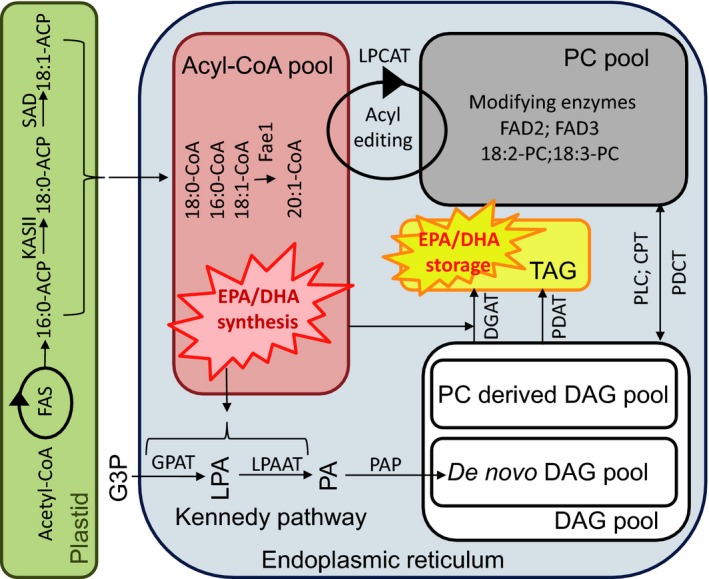
Schematic diagram of the major pathways involved in the production of triacylglycerol (TAG) in seeds. The successful synthesis of eicosopentanoic acid (EPA) and docosahexaenoic acid (DHA), avoiding metabolic bottlenecks, requires the coordinated use of coenzyme A (CoA)‐based desaturase and elongase activities. The incorporation of these long chain omega‐3 fatty acids into TAG then depends on the activities of endogenous TAG assembly pathways. Metabolite abbreviations: ACP, acyl carrier protein; DAG, diacylglycerol; G3P, glycerol‐3‐phosphate; LPA, lysophosphatidic acid; PA, phosphatidic acid; PC phosphatidylcholine, TAG, triacylglycerol. Enzyme abbreviations: CPT, CDP‐choline:diacylglycerol cholinephosphotransferase; DGAT, diacylglycerol acyltransferase; FAD, fatty acid desaturase; FAS, type II fatty acid synthase complex; GPAT, glycerol‐3‐phosphate acyltransferase, KAS, β‐ketoacyl‐ACP synthase; LPAAT, lysophosphatidic acid acyltransferase; PAP, phosphatidic acid phosphatase; PDCT, phosphatidylcholine:diacylglycerol cholinephosphotransferase; PLC, phospholipase C; SAD, Δ9 stearoyl‐acyl carrier protein desaturase.

Seeds contain storage lipids predominantly in the form of TAG, a glycerol backbone onto which three fatty acids are sequentially esterified. The synthesis and assembly of TAG in plants is complex, involving a metabolic network of fatty acid fluxes through multiple subcellular compartments containing alternative pathways to produce different lipid compositions (Figure 1). The reader is encouraged to refer to a number of recent reviews for a more detailed description (Bates *et al*., 2013; Chen *et al*., 2015). Briefly, fatty acids are synthesized in the plastid by a Type II fatty acid synthase complex. A repeated series of condensation, reduction and dehydration reactions adds two carbon units to the extending fatty acid chain. The final products of this cyclical reaction are fatty acids typically 16 or 18 carbons long and attached to an acyl‐carrier protein (ACP). Whilst in the plastid a double bond can be introduced through the action of a Δ9‐desaturase. The ACP moeity is removed by thioesterases and the fatty acids produced in the plastid are then exported (via FAX1; Li *et al*., 2015) to the cytosol, converted to CoA forms and rapidly incorporated into phosphatidylcholine (PC). Further modification by additional desaturation or incorporation of functional groups can then occur. A process of acyl editing (mediated by lysophosphatidylcholine acyltransferases, LPCATs) exchanges fatty acids between PC and the acyl‐CoA pool (Bates *et al*., 2012; Wang *et al*., 2012; Lager *et al*., 2013). Once located in the endoplasmic reticulum (ER), fatty acids are assembled into TAG by a combination of two pathways. The acyl‐CoA‐dependent Kennedy pathway begins with the sequential acylation of glycerol‐3‐phosphate by glycerol‐3‐phosphate acyltransferases (GPATs) and lysophosphatidic acid acyltransferases (LPAATs) using acyl‐CoA to produce phosphatidic acid (PA). This PA can then be dephosphorylated by PA phosphatases to create *de novo* diacylglycerol (DAG). The DAG is then available for two different acyltransferase reactions: diacylglycerol acyltransferases (DGAT) transfer acyl‐CoAs to the *sn‐3* position of DAG to produce TAG; alternatively phospholipid:diacylglycerol acyltransferases (PDAT) transfer the *sn‐2* acyl group of from phospholipids to DAG, forming TAG (Stahl *et al.,* 2004; Dahlqvist *et al*., 2000, Liu *et al*., 2012). The contribution of DGAT and PDAT to TAG synthesis is known to vary between species. In addition to the DAG derived from the Kennedy pathway, experiments have identified a second PC‐derived DAG pool (Bates and Browse, 2011). The interconversion of PC and DAG can occur via a number of different activities; primarily phosphatidylcholine:diacylglycerol cholinephosphotransferase (PDCT; also known as ROD1) which transfers the head group from PC to DAG; other mechanisms might include phospholipase C or the reverse reaction of CDP‐choline:diaclglycerol cholinephosphotransferase (CPT) (Lu *et al*., 2009). Any PC‐derived DAG produced from these activities can then be assembled into TAG via the action of a DGAT and/or PDAT.

## Example 1: Generating Novel Enzyme Activities For The Production of Omega‐7 Unsaturated Oils

Vegetable oils are typically enriched in unsaturated fatty acids that contain a Δ9 double bond, primarily in C18 acyl chains, like oleic (18:1Δ^9^) and linoleic (18:2Δ^9,12^) acids. Using the ‘omega’ nomenclature for unsaturated fatty acids, in which the position of the double bond is determined by counting carbon atoms starting from the methyl end of the acyl chain, the Δ9 double bond of these C18 fatty acids is at the omega‐9 position. This double bond is formed by the activity of the soluble, plastid‐localized Δ9 acyl‐acyl carrier protein (ACP) desaturase, which is most active with stearoyl (18:0)‐ACP (Shanklin and Cahoon, 1998). The availability of a crystal structure for the castor 18:0‐ACP desaturase (Lindqvist *et al*., 1996) along with primary structures of naturally occurring acyl‐ACP desaturases with variant fatty acid substrate chain lengths and regio‐specificities (Cahoon *et al*., 1992, 1994, 1997a, 1998; Schultz *et al*., 1996) enabled the synthetic *in silico* design of acyl‐ACP desaturases with altered activities (Cahoon *et al*., 1997b, 1998; Cahoon and Shanklin, 2000; Whittle and Shanklin, 2001). Among these ‘new’ desaturases were those that were most active as Δ9 desaturases with 16:0‐ACP, rather than 18:0‐ACP, resulting in the production of the omega‐7 unsaturated fatty acid palmitoleic acid (16:1Δ9) (Cahoon *et al*., 1997b, 1998; Cahoon and Shanklin, 2000; Whittle and Shanklin, 2001). The design of Δ9‐16:0‐ACP desaturases could be achieved with as little as one amino acid substitution at the bottom of the active site of the archetypical acyl‐ACP desaturase (Cahoon and Shanklin, 2000).

**Figure 2:**
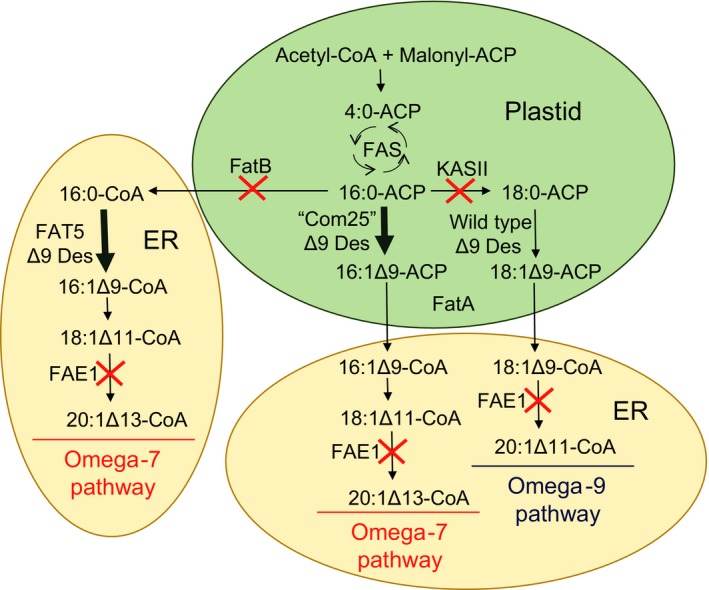
Strategy for metabolic redesign of fatty acid desaturation and biosynthetic pathways for production of omega‐7 monounsaturated fatty acids. Seeds of most oil crops accumulate unsaturated fatty acids derived almost entirely from the omega‐9 desaturation pathway that begins in plastids with the Δ9 desaturation of the fatty acid synthase (FAS) product stearoyl (18:0)‐acyl carrier protein (ACP) desaturase. The rational design of a mutant Δ9 acyl‐ACP desaturase (‘Com25’) with specificity for palmitoyl (16:0)‐ACP enabled the redirection of fatty acid flux for the synthesis of the omega‐7 monounsaturated fatty acid palmitoleic acid (16:1Δ9) in Camelina seeds. Enhancement of 16:0‐ACP substrate pools for the Com25 Δ9 desaturase was increased by RNA interference (RNAi) suppression of genes for the FatB thioesterases that releases 16:0 from ACP and the β‐ketoacyl‐ACP synthase II (KASII) that elongates 16:0‐ACP to 18:0‐ACP. *Caenorhabditis elegans* FAT5 Δ9 desaturase was introduced to obtain additional production of 16:1Δ9 from 16:0‐CoA in the endoplasmic reticulum (ER). RNAi suppression of the gene for the fatty acid elongase 1 (FAE1) β‐ketoacyl‐CoA synthase was conducted to block fatty acid elongation and enhance accumulation of C16 and C18 omega‐7 monounsaturated fatty acids.

The design of a Δ9‐16:0‐ACP desaturase to produce omega‐7 unsaturated fatty acids (e.g. 16:1Δ^9^) rather than omega‐9 unsaturated fatty acids (e.g. 18:1Δ^9^) provided the basis for the recently reported redesign of unsaturated fatty acid biosynthesis in *C. sativa* seeds for the production of oils containing up to 60% omega‐7 mononunsaturated fatty acids (Nguyen *et al*., 2015). Such seed oils occur rarely in the plant kingdom, and instead, omega‐7 monounsaturated fatty acids are present at levels of <2% in most oilseed crops (Nguyen *et al*., 2015). Vegetable oils enriched in omega‐7 monounsaturated fatty acids not only have superior oxidative stability relative to polyunsaturated vegetable oils, such as soybean oil, but they can be used as a feedstock to produce 1‐octene (Rybak *et al*., 2008; Meier, 2009; del Cardayre, 2013). This molecule, currently produced from petroleum, is used as a monomer for the synthesis of low‐linear‐density polyethylene. The feasibility of using a designer desaturase to produce oils rich in omega‐7 fatty acid was first shown in Arabidopsis seeds (Cahoon and Shanklin, 2000). The amounts of omega‐7 monounsaturated fatty acids were increased in a β‐ketoacyl‐ACP synthase II (KASII) mutant defective in elongation of 16:0‐ACP to 18:0‐ACP to increase pool sizes of 16:0‐ACP for the Δ9‐16:0‐ACP desaturase (Cahoon and Shanklin, 2000). This strategy was transferred to *C. sativa* using a second generation the Δ9‐16:0‐ACP desaturase (‘Com25’) with improved kinetic properties with the 16:0‐ACP substrate (Nguyen *et al*., 2010, 2015). The expression of the Com25 desaturase alone under control of a strong seed‐specific promoter increased omega‐7 unsaturated fatty acid content in *C. sativa* oil from 1.4% in wild‐type seeds to 17% in engineered seeds (Nguyen *et al*., 2015). Co‐expression of Com25 with the *Caenorhabditis elegans* FAT5 Δ9‐16:0‐CoA desaturase increased omega‐7 monounsaturated fatty acid levels to nearly 25% and inclusion of RNA interference (RNAi) suppression of KASII further increased amounts of these fatty acids to about 40% of the total fatty acids (Nguyen *et al*., 2015). A final assembly of six transgenes (each under control of a seed‐specific promoter) on two T‐DNAs that included two copies of the Com25 desaturase, the FAT5 desaturase and RNAi suppression cassettes for genes encoding KASII, fatty acid elongase 1 (FAE1) and the FatB 16:0‐ACP thioesterases (Figure 2), yielded >60% omega‐7 monounsaturated fatty acids (Nguyen *et al*., 2015). Key to the additional increases in omega‐7 monounsaturated fatty acids from the levels of about 40% achieved with the Com25, FAT5 desaturase and KASII RNAi combination to levels above 60% was RNAi suppression of the FatB 16:0‐ACP thioesterases. This alteration effectively diverted the release of acyl chains from fatty acid synthase to increase 16:0‐ACP substrates for the synthetic Com25 Δ9‐16:0‐ACP desaturase. RNAi suppression of FAE1 in this strategy blocked elongation of the C18 omega‐7 monounsaturated fatty acid 18:1Δ^11^ (*cis*‐vaccenic acid), resulting in the accumulation of primarily 16:1Δ^9^ and 18:1Δ^11^. Overall, this strategy illustrates how fatty acid synthesis and modification reactions can be tailored and iteratively optimized around a synthetic fatty acid desaturase for the redesign of unsaturated fatty acid synthesis to produce compounds of industrial value.

## Example 2: Metabolic Redesign of Seed Storage Oil Forms

**Figure 3:**
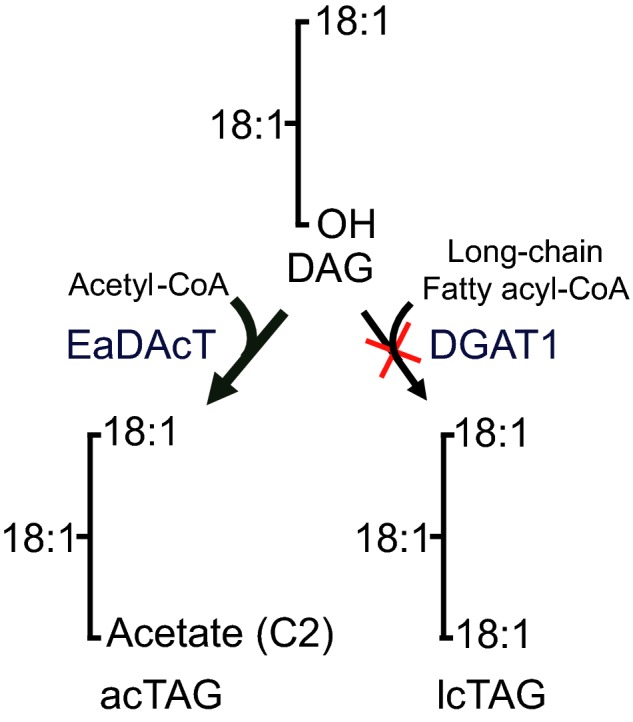
Strategy for redesign of seed storage oil synthesis for production of *sn*‐3 acetyl triacylglycerol (acTAG). Seeds of most oil crops accumulate TAGs generated, in part, through the activity of diacylglycerol (DAG) acyltransferase 1 (DGAT1) that uses DAG and a long‐chain fatty acyl‐CoA as substrates. Through the transgenic expression of a *Euonymus alatus* (burning bush) diacylglycerol acetyl‐CoA transferase (EaDAcT), seed storage oil can be shifted to the production of acTAG, rather than ‘regular’ TAG with long‐chain fatty acids (lcTAG). RNA interference suppression of the gene for DGAT1 relieves the competition for DAG between DGAT1 and EaDAcT to enhance acTAG accumulation. Note that the C2 fatty acid only accumulates at the sn‐3 position of the acTAG.

The primary form of fatty acid storage in seeds of oil crops is TAG. Alternative forms of fatty acid storage occur rarely in seeds, including wax esters in jojoba (*Simmondsia chinensis*) seeds (Naqvi and Ting, 1990) and *sn*‐3 acetyl TAGs in *Euonymus alatus* (burning bush) seeds (Durrett *et al*., 2010 *)*. The latter storage form, referred to as acTAG, is similar to TAG except that acetate (C2) is present at the *sn*‐3 position of TAG instead of a fatty acid (Durrett *et al*., 2010). The result is a vegetable oil with lower viscosity and calorific content than conventional vegetable oils (Durrett *et al*., 2010). These properties make acTAG suitable for specialized lubricant applications and potentially for ‘drop‐in’ (i.e. a direct replacement for petrochemical‐derived compounds) biodiesel, as well as for low‐calorie cooking oils (Durrett *et al*., 2010; Bansal and Durrett, 2016). The synthesis of accTAG in burning bush is catalysed by diacylglycerol acetyltransferase (or EaDAcT), a specialized acyltransferase that uses DAG and acetyl‐CoA (Figure 3) as substrates (Durrett *et al*., 2010). By contrast, the major route of TAG synthesis in typical oilseed crops is catalysed by a Type 1 diacylglycerol acyltransferase (DGAT1) that uses DAG and a fatty acyl‐CoA as substrates to generate TAG with long‐chain fatty acids at the *sn*‐3 position (lcTAG) (Zhang *et al*., 2009). Seed‐specific expression of an EaDAcT cDNA has been used to re‐route DAG flux for the synthesis of acTAG rather than ‘regular’ lcTAG in *C. sativa* and soybean (Liu *et al*., 2015a,b). Using this approach, seed oils containing as much as 70% acTAG (as a percentage of total seed TAG) were obtained in these crops (Liu *et al*., 2015a). *Camelina sativa* lines expressing EaDAcT have subsequently provided a platform for tailoring the composition and content of acTAGs. Seed‐specific RNAi suppression of the *FAD2* gene encoding the Δ12 oleic acid desaturase was used to generate acTAGs enriched in oleic acid (18:1Δ9) and reduced in polyunsaturated fatty acids for improved oxidative stability of acTAG oil (Liu *et al*., 2015b). As described above, EaDAcT and DGAT1 compete for the DAG substrate (Figure 3). This competition creates a bottleneck for increasing acTAG levels in *C. sativa*. To alleviate this bottleneck, seed‐specific RNAi suppression of DGAT1 was introduced into *C. sativa* lines expressing EaDAcT (Liu *et al*., 2015a). The result was an enhancement in the amounts of acTAG up to 85% of the total TAG of *C. sativa* seeds with no apparent effect on seed viability (Liu *et al*., 2015a), implying that these novel (ac)TAGs are recognized as substrates by the endogenous enzymes that catabolize storage reserves to provide energy for seed germination In general, it is essential that there is no impairment to seed germination as a consequence of altered lipid composition. These designer *C. sativa* lines offer additional possibilities for further tailoring the specialty acTAG oils, including introduction of fatty acids into the DAG backbone with chain‐lengths of <C16 to gain further reductions in oil viscosity and enhancing cytosolic acetyl‐CoA concentrations for increased acTAG content. Currently, the use of EaDAcT limits the incorporation of acetyl‐CoA to the sn‐3 position of the nascent TAG molecule. However, future work could focus on how to further increase the accumulation of acetate on the glycerol backbone.

## Example 3: Nutritional Improvement of Oil – The Production of Omega‐3 Fatty Acids

**Figure 4:**
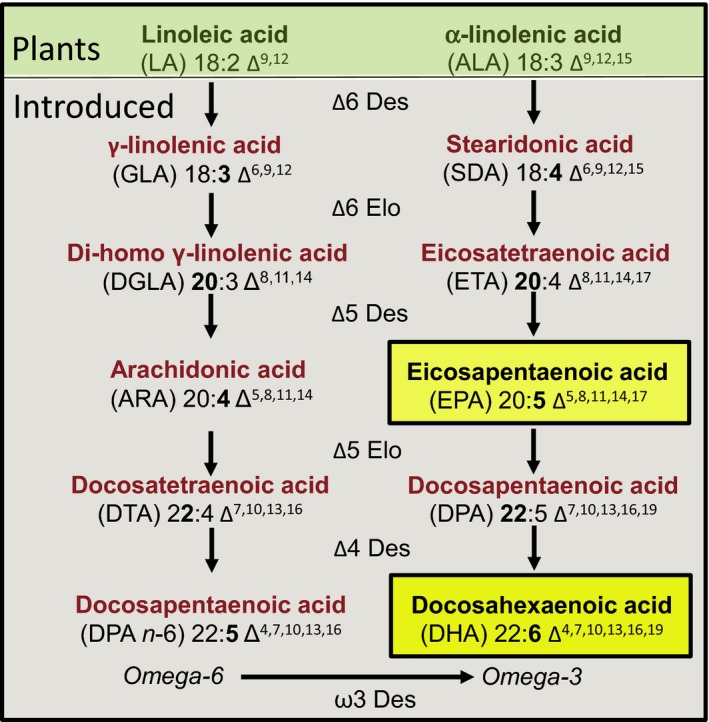
A representation of the Δ6‐pathway for biosynthesis of long‐chain polyunsaturated fatty acids in plants. The substrates linoleic acid (LA) and α‐linolenic acid (ALA) (highlighted in the box at the top) are sequentially converted through the combined activity of separate desaturase (Des) and elongase (Elo) to the target long‐chain omega‐3 fatty acids – eicosopentanoic acid (EPA) and docosahexaenoic acid (DHA). Additional enzymes with omega‐3 desaturase (ω3 Des) activities are also included to enhance the production of EPA and DHA over omega‐6 forms such as arachidonic acid (ARA) via the generic conversation of omega‐6 to omega‐3. An alternative configuration of the pathway (not illustrated) begins with a Δ9‐elongation and two rounds (Δ8, Δ5) of desaturation to generate ARA and EPA.

The successful conversion of native plant fatty acids such as linoleic acid (LA; 18:2Δ^9,12^) and α‐linolenic acid (ALA; 18:3Δ^9,12,15^) to omega‐3 long‐chain polyunsaturated fatty acids (LC‐PUFA) like eicosapentaenoic acid (EPA; 20:5∆^5,8,11,14,17^) and/or docosahexaenoic acid (DHA, 22:6∆^4,7,10,13,16,19^) requires the introduction and coordinated expression of multiple transgenes. Two separate and converging aerobic routes have been identified for the biosynthesis of the C20 LC‐PUFAs arachidonic acid (ARA; 20:4Δ^5,8,11,14^) and EPA (for review see Venegas‐Caleron *et al*., 2010; Haslam *et al*., 2013): (i) the predominant ‘conventional’ Δ6‐pathway which commences with the introduction of a double bond at the Δ6 position, followed by C2 chain elongation and a second desaturation at the Δ5 position in the C20 acyl chain, generating EPA and ARA, and (ii) an alternative configuration (or Δ9‐pathway) which begins with a Δ9‐elongation, and two rounds (Δ8; Δ5) of desaturation (Figure 4; Wallis and Browse, 1999; Sayanova *et al*., 2006; Damude and Zhu, 2007; Zhou *et al*., 2007; Sayanova *et al*., 2011). In lower eukaryotes, the synthesis of DHA requires a further two steps – Δ5 elongation and Δ4 desaturation – whereas in higher organisms, a much more inefficient route involving two cycles of chain elongation to C24, followed by Δ6‐desaturation and beta‐oxidation, is present.

Following the successful reconstitution of LC‐PUFA biosynthetic pathways in yeast (Beaudoin *et al*., 2000; Domergue *et al*., 2003; Meyer *et al*., 2004) the first successful demonstration of EPA production in plants was published by Qi *et al*. (2004), who expressed algal components of the alternative pathway in the leaves of Arabidopsis. The production of EPA in seeds was first described by Abbadi *et al*. (2004), who expressed the conventional pathway in transgenic linseed (*Linum usitatissimum*). Analysis of the lipids in the seeds and leaves of the transgenic plants confirmed the production of moderate levels of EPA but also high levels of C18 Δ6 desaturation products, e.g. γ‐linolenic acid (18:3 ω‐6, GLA) and stearidonic acid (18:4 ω‐3, SDA). Following these two studies many researchers undertook an iterative engineering approach (Kinney *et al*., 2004; Wu *et al*., 2005; Cheng *et al*., 2010), using different gene combinations (combinations of the core ∆6‐pathway with omega‐3 desaturases and ∆12‐desaturases to increase substrate supply) to reconfigure seed lipid synthesis, increase target fatty acid production and reduce unwanted intermediates. However, detailed analyses of the lipids indicated an inefficient transfer of these non‐native fatty acids from the acyl‐CoA pool into extraplastidial phospholipids (Abbadi *et al*., 2004; Sayanova *et al*., 2006). The key bottleneck in the efficient heterologous reconstitution of LC‐PUFA biosynthesis was identified as the so‐called ‘substrate dichotomy’ problem, in that the acyl‐substrates of desaturase and elongase activities utilize different lipid pools, specifically fatty acids either esterified to PC (desaturases) or CoA (elongases). Lack of acyl‐exchange between these different metabolic pools resulted in compromised LC‐PUFA synthesis (Napier, 2007).

The metabolic bottleneck and the resulting accumulation of undesired intermediates could be reduced by the expression of acyl‐CoA‐dependent desaturases, thus enabling desaturation and elongation to occur in the same lipid pool. The utility of this approach was first described in yeast by Domergue *et al*. (2003) and then translated in plants (Hoffmann *et al*., 2008; Petrie *et al*., 2010b; Sayanova *et al*., 2012), demonstrating how the use of acyl‐CoA‐dependent Δ6‐desaturase can enhance the flux of substrate acyl chains through the LC‐PUFA biosynthetic pathway. With the availability of a ‘molecular tool box’ capable of directing EPA/DHA synthesis, a higher‐throughput systematic approach to evaluating gene combinations was then required. Such iterative engineering followed two strategies, the first utilised by Petrie and co‐workers (CSIRO, Australia) made use of *Agrobacterium*‐mediated transient expression of multiple gene combinations in *Nicotiana benthamiana* (Wood *et al*., 2009), allowing the rapid expression of non‐native combinations and evaluation of their efficacy for EPA/DHA synthesis. Although testing was undertaken in leaves, the co‐expression of the Lec2 transcription factor or master regulator essentially ‘reprogrammed’ the leaf to replicate the lipid composition of a seed; a system that permitted the use of seed‐specific (active in response to Lec2 expression) rather than constitutive promoters (Petrie *et al*., 2010a). A second approach, adopted by Heinz *et al*. (University of Hamburg) and Napier *et al*. (Rothamsted Research), was to directly assess gene combinations and their regulatory elements in the seeds of stably transformed plants (namely the oilseed model *Arabidopsis thaliana*; Domergue *et al*., 2005; Ruiz‐Lopez *et al*., 2012, 2013). In combination with the development of lipidomic approaches, this then enabled evaluation of the efficacy of gene combination in the seed. Information from such screening methodologies, which were low‐throughput but information‐rich, enabled rapid progress in construct design, e.g. gene orientation, spacing, and promoter choice for subsequent stable transformation into plants.

Currently, the predominant end‐user of omega‐3 LC‐PUFAs is the aquafeed (fish‐farming) industry. Perhaps counter‐intuitively, marine and salmonid fish species have a very limited capacity to synthesize EPA and DHA and consequently the human nutritional value of the finished product is determined by the diets provided to the farmed fish. To produce the very significant volumes of omega‐3 LC‐PUFAs required by aquaculture necessitates the production of EPA and DHA in agricultural oilseed crops rather than via algal fermentation. The opportunities of scale associated with such crops provide the only economically viable route to generate a fish oil substitute (i.e. about 20% EPA and DHA in total fatty acids) in the required quantities. As an established oilseed crop, *C. sativa* was selected as the best chassis to host the omega‐3 trait and researchers have demonstrated the successful production of a hybrid oil with high levels of EPA and DHA (Petrie *et al*., 2014; Ruiz‐Lopez *et al*., 2014). Analysis of the seed fatty acid composition from stable transgenic lines revealed that the average level of EPA alone was 24 mol%, and for EPA and DHA, 11 and 8 mol% respectively (Ruiz‐Lopez *et al*., 2014). Further experiments using a set of genes encoding the enzymes participating in the alternative Δ9‐pathway (Δ9‐elongating activity and Δ8‐ and Δ5‐desaturases) were evaluated and the accumulation of EPA and the related omega‐3 LC‐PUFA eicosatetraenoic acid (ETA) was observed at levels up to 26 mol% of total seed fatty acids (Ruiz‐Lopez *et al*., 2015). Further field evaluation of the omega‐3 LC‐PUFA trait (EPA + DHA) in *C. sativa* was undertaken and demonstrated (by comparison with replicated glasshouse experiments) the feasibility of producing omega‐3 LC‐PUFAs in the field without any yield penalty (Usher *et al*., 2015). Thus the choice of the oilseed crop *C. sativa* as a host for the successful stable expression of the omega‐3 LC‐PUFA trait was established.

The approaches of CSIRO and Rothamsted have allowed further examination of the strategy for seed lipid redesign. Differences in the accumulations of omega‐3 LC‐PUFA fatty acids illustrated how factors such as selection of the seed‐specific promoter, choice of *C. sativa* variety, enzyme specificity, constructs design/orientation and possibly the site of integration influence outcomes. For example, the choice of Δ5‐elongase defined the extent of EPA accumulation; a highly active Δ5‐elongase will generate DHA at the expense of EPA (Napier *et al*., 2015). Further evaluation of these studies raises questions about the utility of model systems to predict outcomes in crops. Work in Arabidopsis has provided a valuable proof of concept and contributed to the selection of functional gene combinations. However, it would appear that these earlier studies were not reliable proxies for what might be possible in an oilseed crop. For example, work by Ruiz‐Lopez *et al*. in Arabidopsis (2012; 2013) suggested that the maximum levels of EPA accumulation in seeds were about 8%, whereas in *C. sativa* levels of about 24% were observed (Ruiz‐Lopez *et al*., 2014). Evaluation of constructs by Petrie *et al*. also revealed differences in DHA synthesis between Arabidopsis and *C. sativa* (Petrie *et al*., 2012, 2014). In each case, the same construct, when expressed in two different hosts, produced different results. Arabidopsis produced an underestimate of yield potential, at least for omega‐3 production. Clearly then, more thought must be given to the endogenous metabolic context in which a pathway is to be introduced.

## Future Prospects For Plant Lipid Metabolic Design

It is clear from each of the examples above that tools are available for the predictable and reliable synthetic design of seed lipid metabolism. The success of these approaches has required the coordinated and targeted expression of multiple genes and the optimization of enzyme activities. The availability of a adaptable chassis like *C. sativa* will now accelerate progress towards the design and expression of multiple target traits. However, further success can only be achieved by addressing some significant challenges: (i) characterization of the lipid synthesis and assembly network, (ii) the coordinated and targeted expression of multiple genes and functional activities in specific tissue types (compartments), (iii) the assembly of complex transgene cassettes and their targeted insertion into the genome of the host plant, and (iv) the rational design of new synthetic enzyme activities.

Although significant progress has been made in both the synthesis and accumulation of target compounds, identification of key genes associated with lipid synthesis remains elusive. As discussed, many of the enzymes involved in the synthesis and modification of fatty acids and their assembly into TAGs have been well studied and are conserved between most of the major oilseed species. However, comparatively little is known about the genes and assembly of pathways that produce the extensive variation in seed oil composition seen in nature. Even in plants with complete genome sequences, it is difficult to identify all the components of complex lipid metabolic pathways. For example, the use of genome‐wide association studies (GWAS) in Arabidopsis has recently identified new genes associated with lipid synthesis and composition (Branham *et al*., 2015). A GWAS study of 391 wild accessions of Arabidopsis identified four to nineteen regions of interest associated with seed oil composition traits; significantly, two‐thirds of the regions identified contained candidate genes that had never before been implicated in lipid metabolism (Branham *et al*., 2015).

Beyond Arabidopsis, the lack of data is being addressed by large‐scale sequencing projects; comparative transcriptomics is being used to produce large numbers of sequences that can be mined to identify candidate enzymes (Bourgis *et al*., 2011; Troncoso‐Ponce *et al*., 2011; Nguyen *et al*., 2013). Using this approach *C. sativa* oil composition has been tailored towards a superior biofuel and bio‐based lubricant composition (Nguyen *et al*., 2013; Kim *et al*., 2015). Further improvements in oil composition require systems biology approaches to obtain a greater understanding of co‐expression networks, the supply of substrates for oil assembly, regulation of metabolic carbon partitioning and the transcription factors that orchestrate metabolism (Weselake *et al*., 2009).

The application of synthetic biology to lipid metabolic engineering offers the possibility of more rapid assembly of multiple genes for the introduction of complex pathways coupled with transgenes for agronomic traits to generate high‐value products and hasten crop improvement. Particularly appealing are tools such as GoldenBraid 2.0 that enable interchangeable, modular assembly of transgene cassettes (Sarrion‐Perdigones *et al*., 2013, 2014). With these tools, gene combinations for complex pathways can be rapidly evaluated and iteratively improved in an easily transformable plant such as *C. sativa*. In addition, as technologies such as CRISPR/Cas9 advance for targeted genome modification, possibilities exist for the introduction of assembled transgene modules into specific loci in the host crop. It is envisioned that the targeted loci will be in a genome region that is highly transcribed and lacking genes that affect agronomic fitness. Through this approach, the predictability of transgenesis and metabolic outcomes is enhanced, and the need to generate many engineered lines to identify a ‘top‐performer’ is mitigated. In the short term, genome editing techniques can be used to tailor the host crop to optimize pathways. For example, genes for competing pathways can be disrupted to direct metabolic flux toward a desired route. In the case of Example 2, genome editing could be used to ‘knock out’ or reduce DGAT1 activity for enhancement of EaDAcT‐mediated production of acTAG (Figure 3). The efficacy of this approach has recently been demonstrated by use of transcription activator‐like effector nucleases to silence the soybean *FAD2* gene family to create a high‐oleic‐acid seed trait, which may be useful for engineering pathways that require an oleic acid substrate (Haun *et al*. 2014). In addition, genome editing can be used to modify specific amino acids in pathway enzymes to generate altered substrate specificities. For instance, in the case of Example 1 genome editing to mutate one amino acid in the binding pocket of the stearoyl‐ACP desaturase may be sufficient to convert this enzyme into a palmitoyl‐ACP desaturase for omega‐7 fatty acid production (Cahoon and Shanklin, 2000). Overall, the rate and predictability of plant lipid redesign can be achieved by combining synthetic biology tools for modular transgene assembly with targeted insertion of these assemblies into the host genome and by application of targeted genome modification to optimize the host metabolism for the desired pathway.

Much of the discussion in this review has centred on the seed‐specific expression of multiple enzyme activities. The positioning of any new enzyme activity in its correct metabolic context is crucial, as recent advances in imaging mass spectrometry have revealed heterogeneous distribution patterns within maturing seeds (Horn and Chapman, 2014). The observed heterogeneous patterns are strongly supported by precursor–product relationships between acyl groups in PC and TAG molecular species, indicating spatially distinct mechanisms for shuttling acyl chains through different pathways. Any metabolic design for lipid synthesis must recognize this spatial diversity and incorporate it. In the future, structural analysis and a greater understanding of protein–protein interactions may enable the organization of artificial pathways in ordered and regulated arrays within specific compartments of plant cells, thereby matching enzyme activity to the metabolic context. Synthetic scaffolds could offer a modular and highly flexible tool for rationally, post‐translationally organizing/co‐localizing multiple functionally related enzymes in a controllable manner (Pröschel *et al*., 2015).

Lastly, to meet the requirements for novel plant products, it is likely that the metabolic redesign of seed lipid pathways will require new (modified) enzyme activities, e.g. an improved DGAT for the incorporation of EPA/DHA‐CoA into TAG. The discussion above demonstrates the utility of designing a Δ9‐16:0‐ACP desaturase to produce omega‐7 unsaturated fatty acids (e.g. 16:1^Δ9^) in seeds. Codon‐optimization, directed evolution, screening enzyme libraries and the incorporation of non‐natural amino acids all provide ways of improving or generating novel enzymatic activities. These approaches have the potential to produce long‐lasting re‐engineered enzymes with specific properties necessary for versatile bio‐production (d’ Espaux *et al*., 2015). Future metabolic redesign of plant lipid synthesis will require the development of promoters, transcription factors, sensors, switches, enzyme fusions and scaffolds. The collective deployment of these tools will then enable a more complete orchestration of seed lipid metabolism and sustainable yields of target fatty acids.

**Figure 5:**
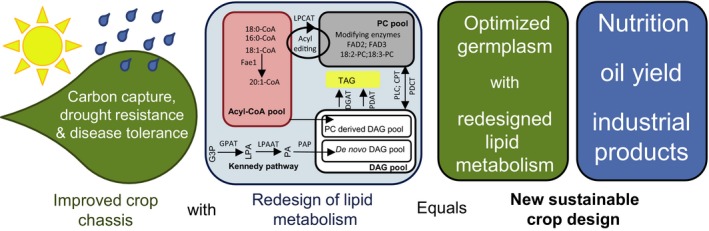
Combining synthetically engineered traits for crop improvement. The synthetic redesign of plant metabolism enables the rapid and predictable engineering of new traits in crops. Bringing these traits in a flexible chassis such as Camelina will in future enable the development of new sustainable crops for agriculture. Abbreviations: CoA, coenzyme A; Fae1, fatty acid elongase 1; G3P, glycerol‐3‐phosphate; GPAT, glycerol‐3‐phosphate acyltransferase; LPA, lysophosphatidic acid; LPAT, lysophosphatidyl acyltransferase; PA, phosphatidic acid; PAP, PAP, phosphatidic acid phosphatase; LPCAT, lysophosphatidylcholine acyltransferase; PC, phosphatidylcholine; FAD, fatty acid desaturase; TAG, triacylglycerol; DGAT, diacylglycerol acyltransferase; PDAT, phospholipid:diacylglycerol acyltransferase; PLC, phospholipase C; CPT, cholinephosphotransferase; PDCT, phosphatidylcholine:diacylglycerol cholinephosphotransferase; DAG, diacylglycerol.

The synthetic redesign of lipid pathways could form part of a greater effort to generate new sustainable crops. Conventional plant breeding is critical for yield improvement, but is limited in the extent of genetic diversity that can be introduced to obtain complex pathways such as those described here. Even most of the commercial biotechnology traits, such as herbicide tolerance and insect resistance, are limited to the expression of one or two transgenes and has resulted in only one‐off improvement of crop production. However, with the development and application of synthetic biology tools which will speed up the design and assembly of large DNA constructs, it will be possible to assemble transgene combinations or ‘stacks’ for output traits such as omega‐3 oils with agronomic traits that underlie crop yields (e.g. carbon, capture, nutrient acquisition and drought/disease resilience) to deliver a step change in crop performance and lower the environmental impact of agriculture (Figure 5). Such an approach is possible in a flexible host chassis such as *C. sativa*, where research has indicated that the expression of photorespiratory bypass is a successful approach towards increasing crop productivity and seed yield (Dalal *et al*., 2015). In future, it may well be possible in *C. sativa* to bring traits such as targeted lipid synthesis together with those for improved crop performance to create a new crop paradigm.

## Conclusions

There are now several successful examples of complex metabolic engineering of oilseeds for a range of useful and valuable traits. In some cases, the engineered plants have undergone field‐trial evaluation, and shown promising stability of the introduced trait under ‘real world’ conditions. Much of this progress has been achieved through a combination of intuitive interventions and several rounds of iteration, in which ‘learning by doing’ plays a critical role. In that respect, a transition to more predicative approaches is both desirable and essential, and new methodologies to further understand the endogenous metabolism, with which transgene‐directed activities must integrate, will provide the underpinning knowledge base by which better interventions might be designed *in silico*. Ultimately, a combination of ‘omic technologies, together with new genome editing capabilities will greatly expand our ability to alter the metabolism of any given chassis. However, the real goal is to deliver such modifications in such a manner that is predictive (and not require multiple iterations), ideally based on computational, rather than biological, interrogation of the system. Even in the intensively studied area of plant seed metabolism, this objective is still some way off, but the examples described in this article demonstrate not only considerable progress but also areas for future study.
